# Plasma Depolymerization of Chitosan in the Presence of Hydrogen Peroxide

**DOI:** 10.3390/ijms13067788

**Published:** 2012-06-21

**Authors:** Fengming Ma, Zhenyu Wang, Haitian Zhao, Shuangqi Tian

**Affiliations:** 1 School of Food Science and Engineering, Harbin Institute of Technology, 73 Huanghe Road, Nangang District, Harbin 150090, China; E-Mails: mfm88492800@163.com (F.M.); zhaoht9999@163.com (H.Z.); tianshuangqi2002@163.com (S.T.); 2 College of Forestry, Northeast Forestry University, 26 Hexing Road Xiangfang District, Harbin 150040, China

**Keywords:** gas discharge, plasma, H_2_O_2_, depolymerization, chitosan

## Abstract

The depolymerization of chitosan by plasma in the presence of hydrogen peroxide (H_2_O_2_) was investigated. The efficiency of the depolymerization was demonstrated by means of determination of viscosity-average molecular weight and gel permeation chromatography (GPC). The structure of the depolymerized chitosan was characterized by Fourier-transform infrared spectra (FT-IR), ultraviolet spectra (UV) and X-ray diffraction (XRD). The results showed that chitosan can be effectively degradated by plasma in the presence of H_2_O_2_. The chemical structure of the depolymerized chitosan was not obviously modified. The combined plasma/H_2_O_2_ method is significantly efficient for scale-up manufacturing of low molecular weight chitosan.

## 1. Introduction

Chitin is the second most abundant natural polysaccharide in nature after cellulose. Chitosan, produced by deacetylation of chitin, has been found to be non-toxic, biodegradable, biofunctional, and biocompatible. It also has strong antimicrobial, hypocholesterolemic, immunity-enhancing and antitumor effects. Chitosan has been applied in many diverse fields, such as food, drug delivery, and biotechnology [1–4]. However, the high molecular weight chitosan (DD > 50%) shows poor solubility in neutral pH aqueous solutions and high viscosity of its solution, which limits its applications [5–7]. In order to improve its solubility and biological, chemical and physical properties, a variety of depolymerization technologies are usually used to prepare low molecular weight chitosan. Therefore, scientists worldwide are focusing on reducing the molecular weight of chitosan without changing its chemical structure by different hydrolysis methods, such as acid [8], hydrogen peroxide [9,10], enzyme [11], ultraviolet light [12], ozone [13], ultrasound [14], radiation [15–17], and so on. In recent years, novel electrotechnologies have been used successfully to prepare low molecular weight chitosan. The effect of pulsed electric fields (PEF) and electrolysis with Ti/TiO_2_-RuO_2_ on the depolymerization of chitosan was demonstrated [18,19].

Non-thermal plasma has emerged as a great prospect for the cold process [20]. Due to its advantages, including operation at atmospheric pressure, no presence of toxic substances, low-temperature, long operative duration, and economical and simple systems [21], the plasma has been applied for various applications, such as pollution control [22], and sterilization [23]. The plasma is generated by electrical discharges in liquid or air. The discharges can produce oxidizing species radicals (H•, O•, OH•, *etc.*) and molecules (H_2_O_2_, O_3_, *etc.*), in addition to shock waves and UV light. These reactive species and physical conditions have sufficient energy levels to break chemical bonds of organic and inorganic substrates, and play a very important role to degrade organic compounds rapidly and efficiently [24–27]. In this paper, the potential of depolymerizing chitosan by plasma from gas discharge of alternating current circuit (AC) is investigated. The synergetic effect of plasma of 0.3% chitosan solution in the presence of H_2_O_2_ (1% and 2%) on the depolymerization of chitosan was studied. In addition, the structure of the depolymerized chitosan was characterized by gel permeation chromatography (GPC), Fourier-transform infrared spectra (FT-IR), UV and X-ray spectra.

## 2. Results and Discussion

### 2.1. Synergetic Effect of Plasma and H_2_O_2_ on Depolymerization of Chitosan

**Figure 1:**
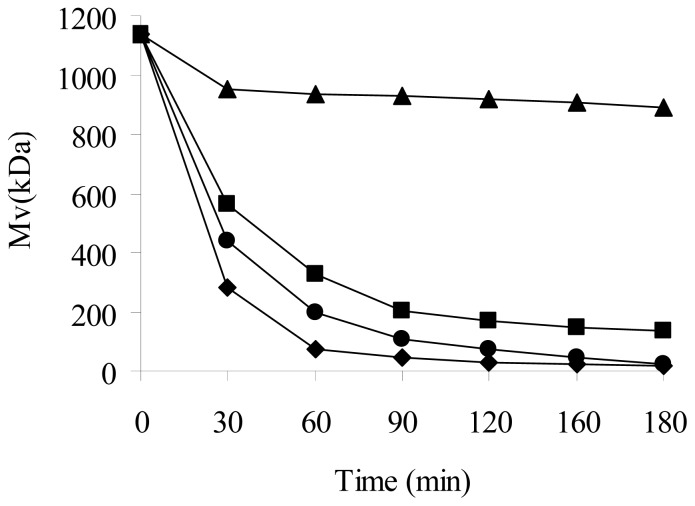
Effect of plasma and H_2_O_2_ on the molecular weight of chitosan. ■: plasma alone; ▲: 2% H_2_O_2_ alone; ●: plasma in the presence of H_2_O_2_ (1%); ♦: plasma in the presence of H_2_O_2_ (2%).

In order to investigate the synergetic efficiency of plasma and H_2_O_2_ on depolymerization of chitosan, two different H_2_O_2_ concentrations, 1% and 2%, were used in the presence of plasma treatment. Figure 1 shows that the combined treatment by plasma and H_2_O_2_ could significantly enhance the depolymerization of chitosan compared to treatment with plasma or H_2_O_2_ alone at room temperature. The higher the concentration of H_2_O_2_, the greater the reduction of the M_v_ occurred at the same treatment time.

When the original chitosan was only exposed to plasma radiation, the M_v_ decreased effectively from 1138.11 ± 0.20 to 134.80 ± 0.23 kDa in 180 min. The rate of the depolymerization of chitosan in the M_v_ was 88.15%.

When the original chitosan was treated with only H_2_O_2_ at room temperature, the M_v_ decreased from 1138.11 ± 0.20 to 888.89 ± 0.18 kDa in 180 min. The corresponding rate of decrease in M_v_ was only 21.90%.

When the original chitosan was treated with plasma combined with H_2_O_2_ (2%), the corresponding M_v_ of chitosan decreased from 1138.11 ± 0.20 to 16.25 ± 0.58 kDa in 180 min. The rate of decrease in M_v_ was 98.57%.

Figure 1 also shows that the M_v_ dropped sharply in the first 60 min and then decreased slowly (60–180 min). This suggests that the high molecular weight chitosans were preferentially depolymerized over the low-molecular-weight chitosans. The depolymerization of chitosan increased with increasing H_2_O_2_ concentration. When H_2_O_2_ concentration was 1% and 2%, the M_v_ in 60 min was 199.72 ± 0.15 and 72.44 ± 0.31 kDa, respectively. The rate of decrease in the M_v_ of chitosan was 82.45% and 93.64%, respectively.

**Figure 2:**
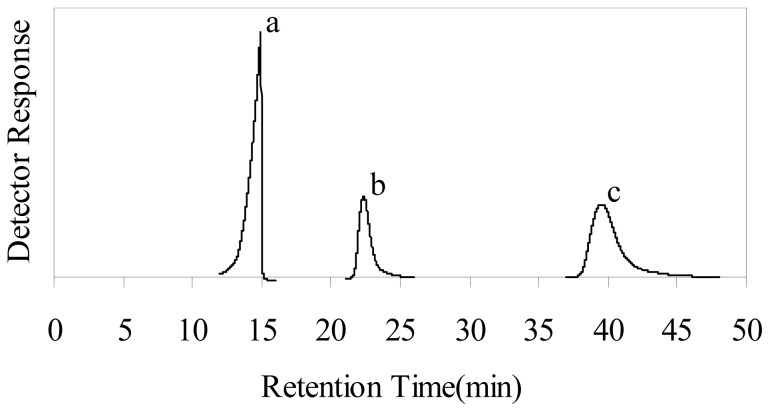
Gel permeation chromatography (GPC) chromatograms of the original chitosan and depolymerized chitosan (depolymerized by plasma in the presence of 2% H_2_O_2_ for different times): (**a**) original chitosan; (**b**) 60 min; (**c**) 180 min.

To further confirm the effect of plasma depolymerization of chitosan, GPC was used for qualitative evaluation of the reduction in molecular weight of chitosan and its distribution. Figure 2 shows the GPC elution curves of original chitosan and plasma chitosan in the presence of 2% H_2_O_2_ after 60 and 180 min. The large molecular weight molecules of large size appeared at low retention time and the lower molecular weight molecules of small size appeared at high retention time. This observation implies that some depolymerization had already occurred. Such a change is reckoned with the expected dominance of chain scission events in the depolymerization of chitosan. The plasma also causes a broadening of the overall distribution, which is diagnostic of the increase of the polydispersity. Therefore, the GPC chromatograms in Figure 2 confirmed the depolymerization effect of plasma on chitosan in the presence of H_2_O_2_.

### 2.2. FT-IR Spectral Analysis

**Figure 3:**
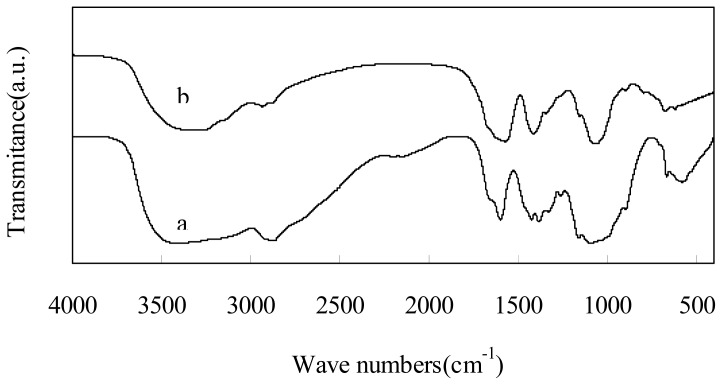
Fourier-transform infrared spectra (FT-IR) spectra of chitosan: (**a**) original chitosan; (**b**) depolymerized chitosan by means of plasma in the presence of 2% H_2_O_2_ after 180 min.

Figure 3 shows the FT-IR spectra of the original chitosan and depolymerized chitosan by plasma treatment with 2% H_2_O_2_ after 180 min. The spectrum of the depolymerized chitosan was similar to that of the original one. The broad absorbent band centered at 3396 cm^−1^ was characteristic of the stretching vibration of –OH and –NH_2_ [28]. The band near 2960–2850 cm^−1^ corresponds to C–H stretching of the alkyl substituent [29]. The bands at 1597 and 599 cm^−1^ were attributed to the binding vibrations of the amido groups [30]. The bands in the range 1158–895 cm^−1^ are assigned to the characteristics of β-D-(1→4) glycosidic bond in chitosan [31,32]. The result of FT-IR spectra suggested that there was no significant difference between the chemical structure of the depolymerized and original chitosan. In addition, the band at 1735 cm^−1^, which assigned to the carboxyl group, was not obvious, indicating that the carboxyl group of chitosan was not formed during depolymerization. In conclusion, the original monomeric structure of chitosan is retained in the depolymerized chitosan.

### 2.3. UV Spectral Analysis

**Figure 4:**
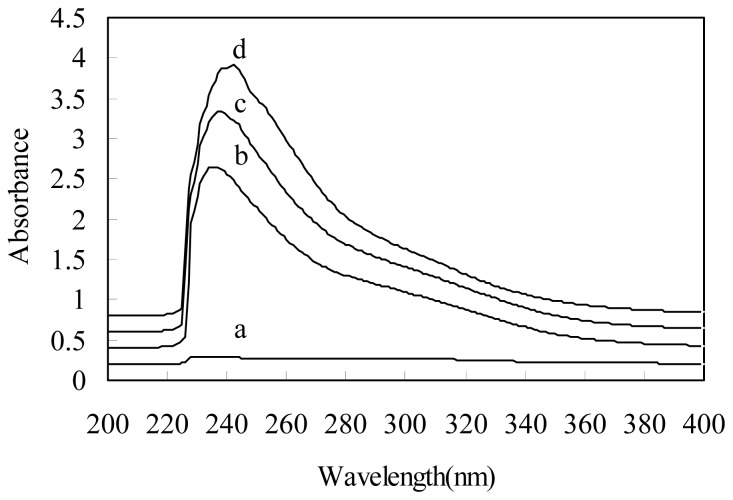
UV spectra of the original chitosan and depolymerized chitosan (depolymerized by plasma in the presence of 2% H_2_O_2_ for different times): (**a**) original chitosan; (**b**) 60 min; (**c**) 120min; (**d**) 180 min.

We studied the stability of the depolymerized chitosan by investigating the UV spectra. Figure 4 shows the UV spectra of the original chitosan and depolymerized chitosan after plasma treatment with 2% H_2_O_2_ at different times. For the treated chitosans, a new absorption band at 240 nm was observed, which was ascribed to carbonyl groups [19,33]. The relative absorption intensity of the peaks increased with increasing treatment time. This result indicates that the carbonyl groups might be formed during depolymerization.

### 2.4. X-ray Analysis

**Figure 5:**
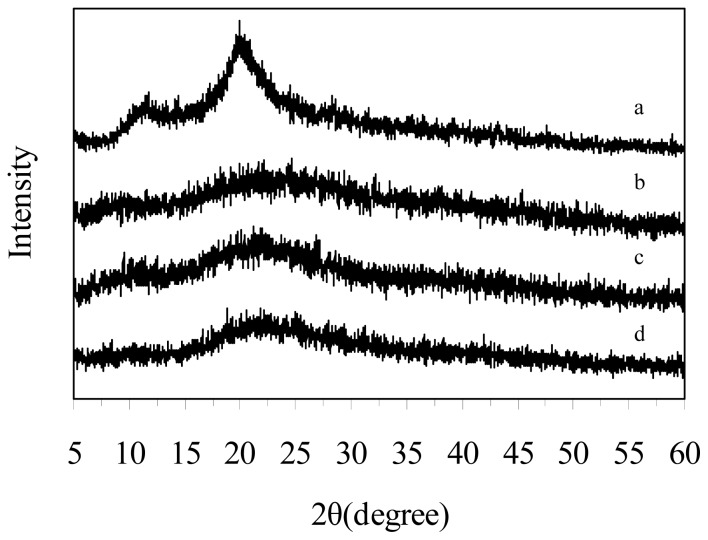
X-ray diffraction (XRD) patterns of the original chitosan and depolymerized chitosan (depolymerized by plasma in the presence of 2% H_2_O_2_ for different times): (**a**) original chitosan; (**b**) 180 min; (**c**) 120 min; (**d**) 60 min.

Figure 5 shows the X-ray diffraction patterns of original chitosan and depolymerized chitosan (60 min, 120 min, 180 min). For the original chitosan, the treatment conditions were the same as the depolymerized chitosan except for the plasma; there were two characteristic peaks at 10.42 and 19.6. Compared with the original chitosan, the depolymerized chitosan sample prepared by plasma combined with H_2_O_2_ had only the peak at 19.6 with less intensity and became amorphous. The present results indicate that the synergistic depolymerization of chitosan by plasma/H_2_O_2_ caused destruction of the crystal structure.

## 3. Experimental Section

### 3.1. Materials

Chitosan was supplied by Sinopharm Chemical Reagent Co., Ltd, China. The degree of deacetylation (DD) of chitosan was not less than 90%. The viscosity-average molecular weight (M_v_) was about 1138.11 ± 0.20 kDa. All other chemicals, including H_2_O_2_, acetic acid (CH_3_COOH), sodium acetate (CH_3_COONa), NaOH and ethyl alcohol were of reagent grade obtained from Sinopharm Chemical Reagent Co., Ltd, China. All solutions were prepared using distilled water.

### 3.2. Plasma Treatment Experiments

**Figure 6:**
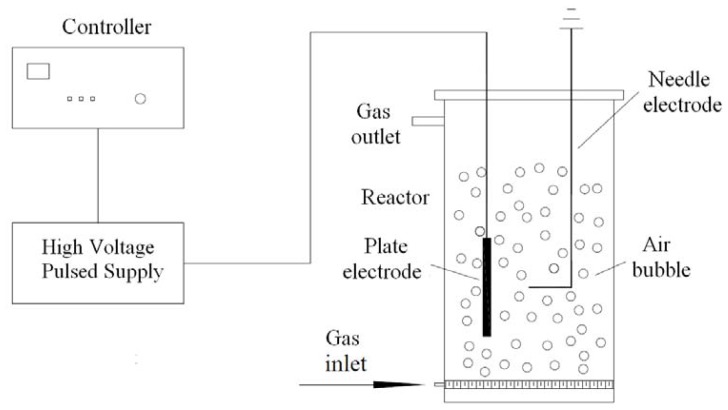
The schematic of plasma experimental apparatus.

A home built apparatus for the plasma treatment, as shown in Figure 6, includes a pulsed high voltage generator with AC and a treatment chamber (100 mL) with needle-plate electrodes. The needle electrode was a stainless steel needle (diameter 1 mm). The plate electrode was a stainless disk (diameter 40 mm). The high voltage pulse generator provided a discharge for a few microseconds. During the treatment, the peak pulse voltage is 60 KV, the power is 350 W, the distance between the electrodes is 2 mm and the treatment time is 180 min. When the pulsed power system was activated, the generator supplied the electrical discharges, which established an electric field across the sparged liquid. Thus the dispersed air bubbles in the liquid are ionized, and the plasma is obtained.

### 3.3. Preparation of Depolymerized Chitosan

Chitosan powder was dissolved into 1% (*v*/*v*) acetic acid aqueous solution. The final concentration of chitosan was 0.3 (wt.%). The 80 mL chitosan solution was treated by plasma in the presence of H_2_O_2_ at room temperature.

### 3.4. Separation (Fractionation) of Water-Soluble Chitosan

After depolymerization, the solution was neutralized with 2 M NaOH solution to pH ~7.5, and then twice the volume of the solution of absolute ethanol was added to precipitate chitosan molecules from dispersions, and then the solid product was filtrated, and washed with ethanol. Finally, the solid product was dried in an oven at 60 °C and the powder of water soluble chitosan was obtained.

### 3.5. Characterization

The molecular weight of chitosan was determined by the viscometric method [31]. The relative viscosity, *η_r_*, of chitosan was measured using an Ubbelohde capillary viscometer (Shanghai Longtuo Instrument Co., Ltd., China) in a constant-temperature water bath at 25 ± 0.5 °C as the mean of three replicates from the same chitosan solution. The intrinsic viscosity, [*η*], was calculated according to Equation (1):

(1) $$[\eta ]=\frac{\left(\eta_{sp}+3\;\text{ln }\eta_{r}\right)}{4c}$$

where *η_sp_* is the incremental viscosity, *η_sp_* = *η_r_* − 1, *c* is the concentration of chitosan (g/mL). Then, the M_v_ of chitosan was calculated based on the Mark–Houwink Equation (2:

(2) $$[\eta ]=k\times {\left(M_{v}\right)}^{a}$$

The value of *k* and *α* is 6.589 × 10^−3^ and 0.88, respectively [34].

GPC was used for qualitative evaluation of the reduction in molecular weight of chitosan on a GPC instrument (Agilent 1100) equipped with a refractive index detector. GPC measurement was carried out at 30 °C using connected columns (79911GF-083 and 79911GF-084) with 0.2 M CH_3_COOH/0.1 M CH_3_COONa solution as an eluent at a flow rate of 1 mL/min.

FT-IR spectrum was measured in powder form on Bruker Vector 22 spectrophotometer (Germany) using KBr pellets in the range of 400–4000 cm^−1^.

UV-Vis spectrum was taken on a TU-1810 (Beijing Purkinje General Instrument Co., Ltd., China) in the range of 200 to 400 nm.

X-ray powder diffraction (XRD) patterns of the depolymerized chitosan fraction were measured on a Rigaku D/max-2200 X-ray diffractometer using a CuKα target at 30 KV and 30 mA at 25 °C. The relative intensity was recorded in the range (2*θ*) of 5–60° with a scanning rate of 0.02°/s.

## 4. Conclusions

Chitosan was efficiently depolymerized by plasma treatment in the presence of H_2_O_2_.The results of GPC analysis showed that the molecular weight of chitosan decreased with the increase of the treatment time. The FT-IR spectra indicated no obvious modification of chemical structure of chitosan before and after depolymerization. The UV spectra suggested that carbonyl groups might be formed during the depolymerization. The results of XRD confirmed this claim that the reduction in molecular weight of the resulting chitosan led to the transformation of the crystal structure. Therefore, plasma treatment in the presence of H_2_O_2_ is a potentially applicable technique for the production of low-molecular-weight chitosan.
